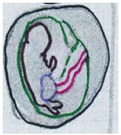# Role of Ultrasound in Body Stalk Anomaly and Amniotic Band Syndrome

**DOI:** 10.1155/2016/3974139

**Published:** 2016-09-04

**Authors:** Madhavilatha Routhu, Sreedevi Thakkallapelli, Prashanthi Mohan, Nadeem Ahmed

**Affiliations:** ^1^Department of Radiology, MGM Hospital, Warangal, Telangana, India; ^2^Department of Surgery, Mamata Medical College, Telangana, India

## Abstract

Body stalk anomaly (BSA) and amniotic band syndrome (ABS) are rare similar fetal sporadic polymalformative syndromes of unknown etiology, though there are certain differences between them. BSA is a combination of developmental abnormalities involving neural tube, body wall, and the limbs with persistent extra embryonic coelomic cavity. ABS is characterized by the presence of thin membrane-like strands attached to fetal body parts and causing constrictions and amputations. This is a cohort study involving 32,100 patients who were referred for routine antenatal ultrasound scan. The data was entered prospectively into a computer database. The duration of study was 3 years. In our study, ultrasound examination in 86 patients demonstrated ventral wall defects, craniofacial defects, and spinal and limb deformities as isolated or combined abnormalities. In those, 10 patients were suspected/diagnosed as BSA/ABS including a twin of a dichorionic diamniotic gestation. The typical features of body stalk anomaly can be detected by ultrasound by the end of the first trimester, which is important for the patient counselling and management. We are presenting these rare conditions and highlighting the importance of early sonographic imaging in diagnosing and differentiating them from other anterior abdominal wall defects.

## 1. Introduction

BSA results from the breakdown of ectodermal placode involving the early embryonic folding process. This syndrome was first described by Van Allen et al. in 1987 [1] and they mentioned 3 essential features:

 Exencephaly or facial clefts or encephalocele.
 Thoraco or abdominoschisis.
 Limb defects.

Incidence ranges traditionally in literature as one in 14,000–22,000 pregnancies [2, 3]. Others reported higher incidence from 1 in 4000 pregnancies [4] to 1 in 7500 pregnancies [5]. Most of them are diagnosed by ultrasonography in the prenatal period and are not continued till birth. The incidence at birth is much lesser and is about 11/428,599 births [6] or about 0.2-0.3 per 10,000 births [7, 8]. Usually it has a normal karyotype but body stalk anomaly may also be associated with placental trisomy 16 or maternal uniparental disomy 16 [9]. The chance of recurrence is very low in subsequent pregnancies. There is no correlation with parents' age or fetal gender [1, 10].

The ultrasonographic milestone of BSA consists of thoracoabdominal defects, spinal cord abnormalities, positional limb deformities, abnormalities of umbilical cord and membranes [10], and persistent extra embryonic coelomic cavity. Ultrasound appearance of ABS is usually thin membrane-like strands criss-crossing the amniotic sac and attached to fetal body parts. The most common finding is constriction rings which can be demonstrated on entangled body parts and are often associated with distal lymphedema. Associated fetal anomalies are usually present and may be severe. These include asymmetric craniofacial clefts, abdominal wall disruptions, and limb deformities. Any part of the body may be involved and the characteristic anomalies can be described as restrictions, constrictions, dissection, and amputations. Although some of the features are similar in both conditions, there are distinctive characteristics in BSA, like marked scoliosis, evisceration of abdominal contents into the extraembryonic coelomic cavity, and a shortened umbilical cord [11]. Limb amputations are not typically found in BSA where the abnormality is predominant in the thoracic/abdominal wall.

## 2. Materials and Methods

In this cohort cross-sectional study, we prospectively entered the data of 32,100 pregnant women referred for routine antenatal scans at 2 centers in Warangal, from February 2013 to January 2016, into computer database. In that, 10 patients were diagnosed as BSA/ABS. Ethical committee approval was taken to conduct the study. Written informed consent was obtained from patients who participated in this study. All the scans were done on Voluson E8 (GE Healthcare), IU 22 (Philips Healthcare), and Esoate My Lab 40 ultrasonographic machines. A general sonographic examination was performed to search for fetal abnormalities and was followed by detailed evaluation including color Doppler study. Nuchal translucency (NT) measurements were performed in those cases presented between 11 and 13 weeks and 6 days of gestation and were measured in a true midsagittal section with the fetus in a neutral position. During the scan, more than two measurements were taken and the largest one was entered into the database. Demographic data, biometric parameters, and sonographic findings of these examinations were stored in a computer database.

## 3. Results

This study involved 32,100 pregnant women who were referred for routine antenatal scans from February 2013 to January 2016. In those, isolated defects were observed as follows: 22 cases of anterior abdominal wall defects, 18 cases of acrania/anencephaly, 24 cases of kyphoscoliotic deformity with and without myelomeningocele, and 12 cases of limb deformities and multiple defects were seen in 10 patients suspected/diagnosed as BSA/ABS. This study is based on those 10 cases and differentiating them from the other abnormalities.

In the above 10 cases, 2 cases were of ABS and 8 of them were BSA including one twin of dichorionic diamniotic pregnancy. Herniation of abdominal contents into coelomic cavity was noted in 8 cases (80%). Thoracic defect with herniation of heart was seen in 5 cases (50%). Cranial abnormalities were detected in 3 cases (40%). Limb abnormalities were present in 7 cases (70%). Spinal deformity was noted in 6 cases (60%), amniotic bands were noted in 4 cases (40%), and short umbilical cord was noted in 4 cases (40%) (Tables 1 and 2). In our cases, NT measurements were performed in 6 cases before 14 weeks of gestation and 5 (83%) fetuses presented with an increased NT (>3 mm). Van Allen in a study of a series of fetuses with BSA found that major structural defects included limb defects (95%), marked scoliosis (77%), internal organ malformation (95%), craniofacial defects (56%), and limb defects including club foot (32%). Body defects were central feature of BSA. In another large series by Van Allen et al., body defects were found in 96% of cases. The involvement of both abdomen and thorax was a more common feature as compared to involvement of either abdomen or thorax solely.

## 4. Discussion

There is no clear etiology of BSA. The theories included in the literature are early amnion rupture [13, 14], vascular disruption [1, 11], and embryonic maldevelopment [15–18].

The first and most accepted theory is an early rupture of the amnion before there is an obliteration of the coelomic cavity. Torpin [13] suggested that the primary rupture of the amnion induces the creation of fibrous bands from the chorionic surface, which entraps the fetal body parts. More recently, different studies have failed to demonstrate any evidence of amnion rupture and fibrotic bands in the presence of body stalk anomaly and therefore challenged the validity of this theory of early amniotic rupture [19–22]. In our study, 4 cases showed amniotic bands which support the above theory.

Van Allen et al. [1, 11] suggested early generalized compromise of embryonic blood flow during the first 4–6 weeks of gestation, leading to failure of closure of the ventral body wall and persistence of the extraembryonic coelomic cavity.

Early embryonic defect as a cause of BSA was initially suggested by Streeter in 1930. Since then, other authors have supported this theory [5, 15, 16, 19, 20, 23, 24]. During the 5th week of gestation, the flat trilaminar embryo is transformed into a cylindrical fetus by a parallel set of four contiguous body folds (cephalic, caudal, and both lateral folds).

Maldevelopment of each of the four folds results in a distinct constellation of anomalies [18]. Abnormal cephalic folding may produce a form of pentalogy of Cantrell, lateral fold defects may result in omphalocele, and aberrant caudal folding may create any or all of the abnormalities of cloacal exstrophy. Body stalk anomaly is supposed to be due to faulty folding in all three axes with persistence of the extraembryonic coelomic cavity. The various malformations associated with body stalk anomaly depend on the degree of aberrant development of each of the four folds.

In our study, 6 out of 10 cases are probably explained by this theory (Figure 11).

Vasoconstrictive agents like cocaine and nicotine may also cause BSA. In our cases, no risk factors or teratogenic factors could be identified.

Few authors [19, 24] described two phenotypes of BSA/ABS according to the placental attachment to body parts:

1. Placentocranial attachment: craniofacial defects and amniotic bands or adhesions are seen which are related to early vascular disruption as elaborated by Van Allen et al. There may be upper limb defects with thoracoschisis or exencephaly/encephalocele.
2. Placentoabdominal adhesion causes lower limb defects, internal organ defects like absent diaphragm, bowel atresia, renal dysplasias, urogenital anomalies, anal atresia, short umbilical cord, and persistent extraembryonic coelomic cavity. It is related to intrinsic embryonic maldevelopment.

The ultrasonographic findings in BSA are

1. evisceration of abdominal content which forms a complex mass covered with membrane floating in the persistent extra embryonic coelom;
2. short umbilical cord which may be adherent to the eviscerated complex mass;
3. internal organ malformation like abnormal mesodermal development;
4. increased nuchal thickness.

Saadi et al., 2007, reported anterior abdominal wall defects with herniation of abdominal contents, kyphoscoliosis, and skeletal defects in upper limb [25].

Martínez-Frías, 1997, in their study, reported the presence of body wall defects with evisceration of thoracic and abdominal organs and other congenital abnormalities with or without limb defects and suggested the term body wall complex [26].

**Figure 9:**
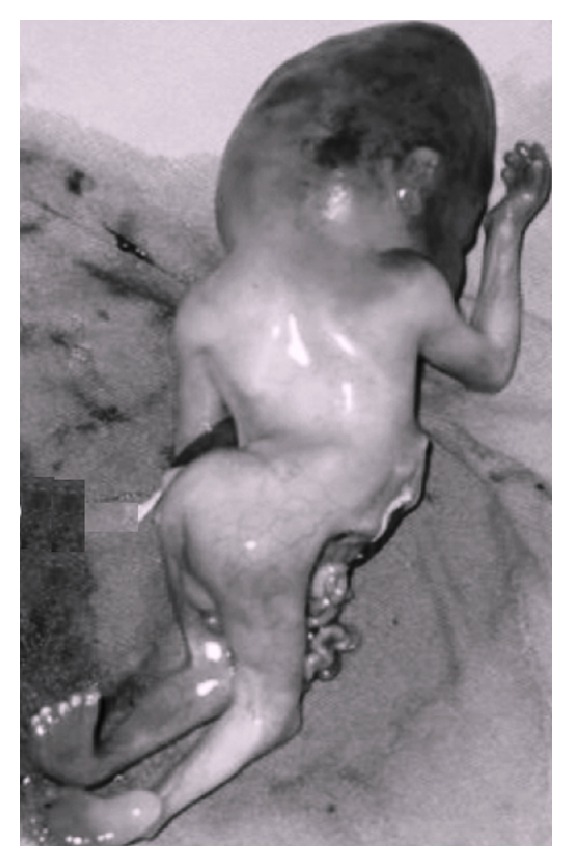
Limb body wall complex.

**Figure 10:**
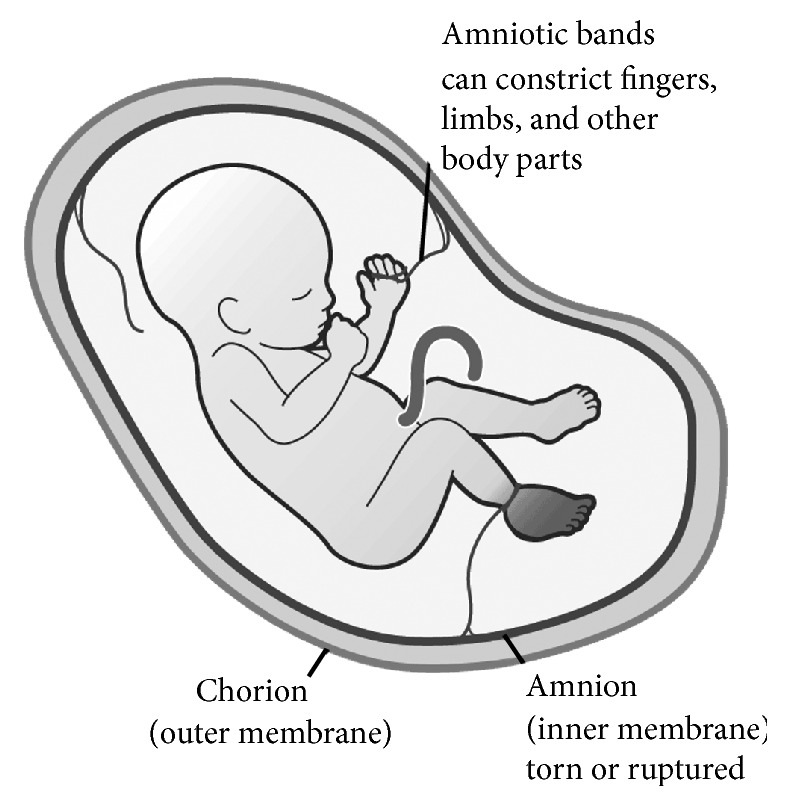
Amniotic band syndrome.

**Figure 11:**
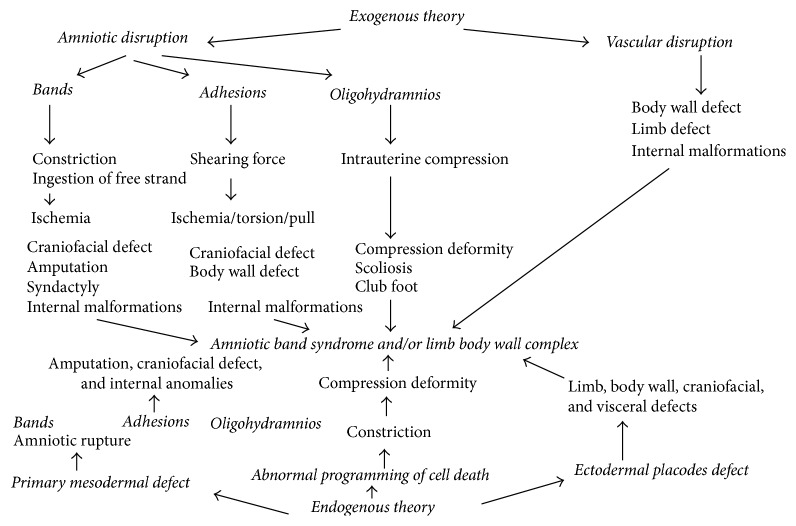
Pathways of body stalk anomaly/amniotic band syndrome arising from different primary events (modified model of Luebke et al. [12]).

Daskalakis et al., 1997, estimated the incidence of BSA as 0.21 to 0.31 cases per 10,000 births [5]. Luehr et al., 2002, reported a higher incidence of 3.3 : 10,000 births probably related to teratogenic causes like tobacco, alcohol, and certain drugs [27]. Some of the research studies showed the connection of BSA with anticonvulsants and cocaine teratogenicity [28]. Hunter and colleagues, 2011, suggested aetiology similar to ectopia cordis, gastroschisis and bladder exstrophy. They suggested that the Limb deficiency may be a secondary complication of primary embryological defect and occurs at 6–10 weeks of gestation [29]. Bugge, 2012, has already mentioned that small umbilical cord and kyphoscoliosis are almost invariably present in BSA [30]. Hartwig et al., 1989, studied four fetuses of BSA. They made it synonymous with ABS (amniotic band syndrome) [16]. Figures 9 and 10 show BSA and ABS (https://fetus.ucsfmedicalcenter.org/amniotic-band-syndrome). The differential diagnosis for BSA is other anterior abdominal and thoracic wall defects like omphalocele, gastroschisis, ectopia cordis, pentalogy of Cantrell, and OEIS (Table 3). Omphalocele is diagnosed when there is supraumbilical defect with herniation of liver, bowel loops, and sometimes urinary bladder. Herniated structures are covered by peritoneum. In gastroschisis, paraumbilical defect is present more commonly on left side with free floating bowel loops in the amniotic cavity. It is a benign condition. OEIS includes infraumbilical abdominal wall defect, exstrophy of bladder or absent bladder, imperforate anus and spinal dysraphism, and a sacral myelomeningocele. Pentalogy of Cantrell is a rare malformation characterized by lower sternal defects, anterior diaphragmatic defects, defect in the pericardium, cardiac ectopia, intracardiac anomalies, and a midline supraumbilical abdominal wall defect. The cause may be related to sex chromosome and trisomy 13.

## 5. Conclusion

For practical purposes, both body stalk anomaly and ABS are similar, being associated with a normal karyotype and sharing a grim prognosis. They appear to be pathogenetically related.

They can be divided into two phenotypes. Placentocranial type leading to amniotic band syndrome and placentocaudal type causing body stalk anomaly (short umbilical cord syndrome). Since BSA is a lethal condition, early diagnosis and its differentiation from other abdominal wall defects are needed. Our study illustrates the value of NT scan at 11–13 weeks and 6 days for early detection and proper diagnosis of these rare conditions. If scan is delayed, most of these fetuses abort spontaneously and exact diagnosis is missed. The diagnosis is important, as these conditions are sporadic and thus karyotyping can be avoided.

As there is no risk of recurrence, the early diagnosis enables reassuring the patient regarding future pregnancies. Twin gestation with one affected fetus may be continued and the management should be focused on the unaffected twin.

## Figures and Tables

**Figure 1:**
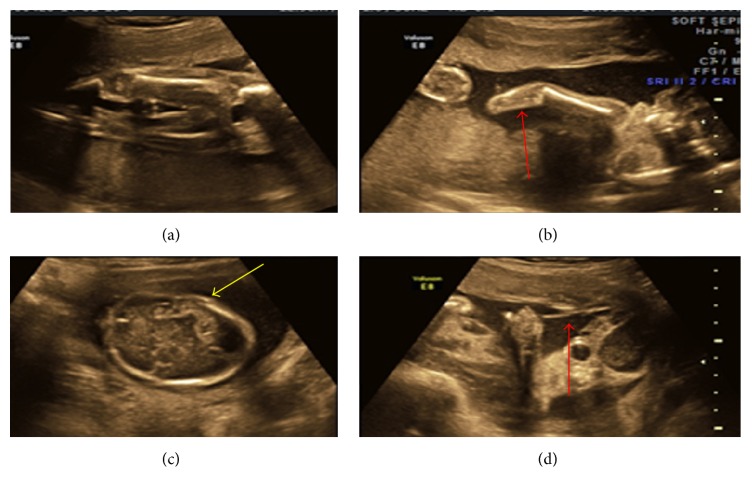
(a) Showing normal lower limbs, (b) showing amputated fore arm with single bone, (c) showing deficient skull vault, and (d) showing amniotic band.

**Figure 2:**
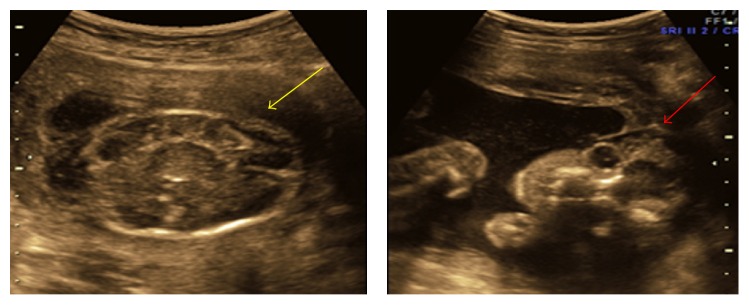
Deficient skull vault with membrane covering brain parenchyma.

**Figure 3:**
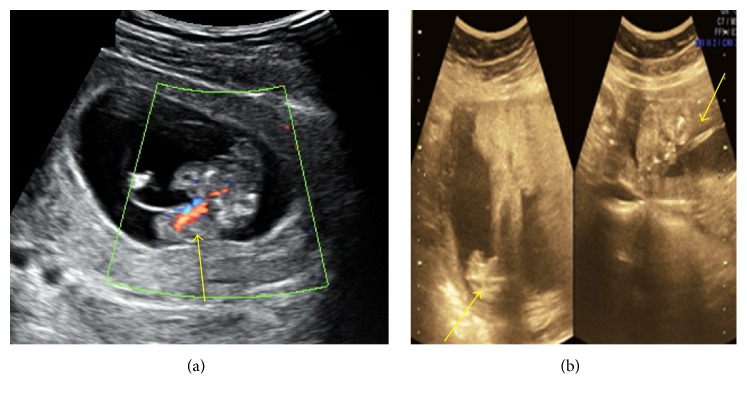
(a) Showing herniated liver in extra embryonic coelomic cavity. (b) Showing short lower limb with club foot and amniotic band.

**Figure 4:**
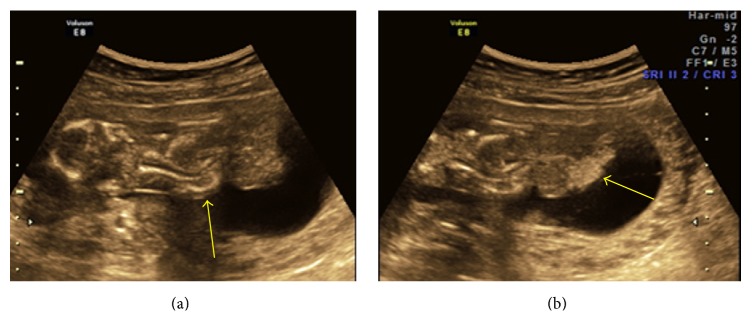
(a) Arrow indicates kyphoscoliosis. (b) Herniation of liver and bowel loops noted in extra embryonic coelomic cavity.

**Figure 5:**
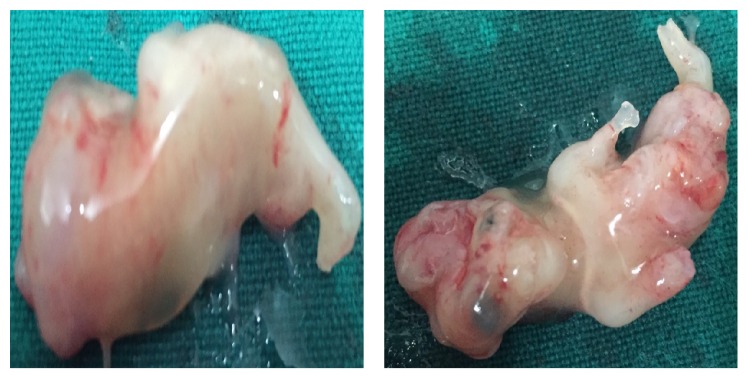
Showing aborted fetus of 11-12 weeks.

**Figure 6:**
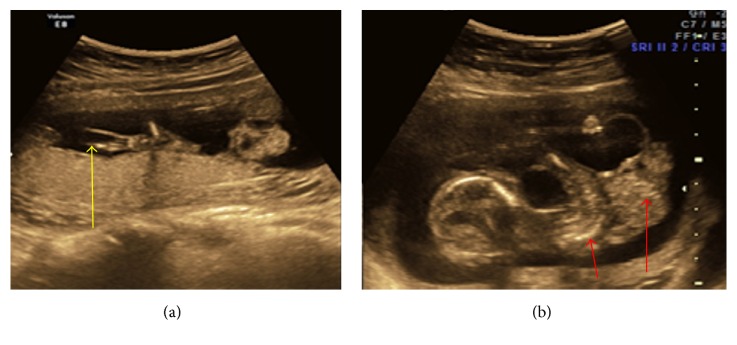
(a) Amniotic bands and (b) kyphoscoliotic deformity with herniation of abdominal content into the coelomic cavity.

**Figure 7:**
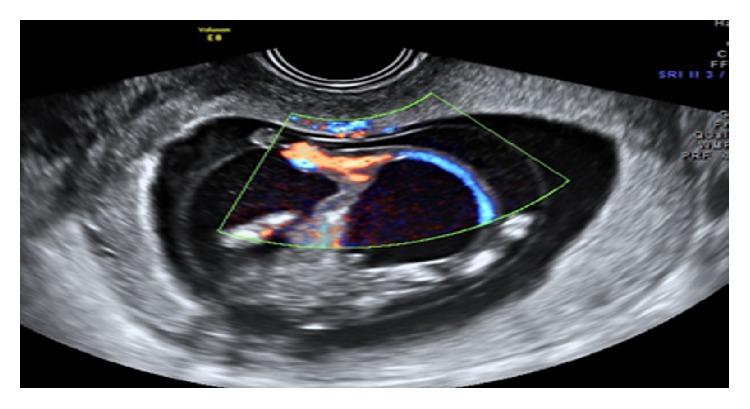
Short umbilical cord with herniation of abdominal contents including urinary bladder.

**Figure 8:**
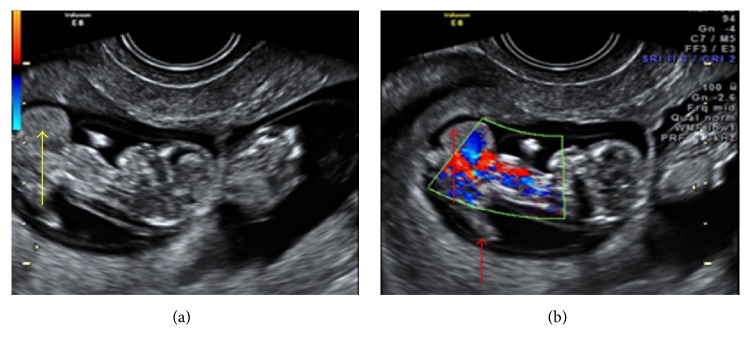
(a and b) Twins with LBWC in one fetus showing herniation of liver in extra embryonic coelomic cavity and abnormally placed lower limbs.

**Table 1:** Summary of major characteristics of the cases. +: present in the case. −: absent in the case. NI: no information.

Manifestations	Case 1	Case 2	Case 3	Case 4	Case 5	Case 6	Case 7	Case 8	Case 9	Case 10
Digital amputation	+	+	−	−	−	−	−	−	−	−
Cranial/CNS defect	+	−	−	−	+	+	−	−	−	−
Limb deficiency/amputation	−	+	−	−	−	−	−	−	−	−
Thoracic defects	−	−	−	−	+	+	−	+	+	+
Body wall defects	−	−	+	+	+	+	+	+	+	+
Short cord	NI	NI	−	−	+	−	+	+	−	−
Spinal deformity	−	−	+	+	+	+	−	+	−	+
Limb deformity	+	+	+	+	−	−	+	+	−	+
Amniotic bands	+	+	+	−	−	+	−	−	−	−
Gestational age in weeks	21-22	31-32	23 ± 1	15-16	10-11	11 ± 1	12-13	12-13	13 ± 1	12 ± 1

**Table 2:** GAD, gestational age at diagnosis; G/P, gravidity/parity; MA, maternal age; NT, nuchal translucency; TOP, termination of pregnancy.

Case	MA	GAD	Abnormal sonographic findings	Autopsy findings
1	22	22 weeks	Deficient skull vault above the orbits anteriorly and occiput posteriorly. Brain parenchyma covered by membrane. Short right forearm with single bone and hand bones could not be made out (Figures 1 and 2).	Extensive irrational asymmetric deformation and disruption. Amputated calvarium, proptosis, right upper limb with short humerus, and short forearm, right elbow joint fused (ulnar club hand), amputation of multiple digits of both hands, syndactyly of first four toes of left foot, and amnion bands confirmed the diagnosis of amniotic band syndrome

2	24	32	Absent right lower limb and short left femur with constrictive ring at lower thigh. Normal left leg bones (live female baby).	

3	22	23-24	Anterior abdominal wall defect with herniation of bowel loops and liver. Persistent extra embryonic coelomic cavity. Short spine with kyphoscoliotic deformity. Bilateral short legs with club foot. Evidence of amniotic bands was noted (Figures 3(a) and 3(b)).	Parents not accepted

4	20	15-16	Abdominal wall defect with herniation of liver and bowel loops. Lower fetus was placed in the coelomic cavity. Abnormally positioned lower limbs, kyphoscoliotic deformity of spine, and multiloculated cystic area were noted at neck region (Figure 4).	Not done

5	24	11 ± 1 WK	Large thoracoabdominal wall defect with evisceration of abdominal contents and heart were noted within the extraembryonic coelomic cavity. Herniated organs are connected to the placenta by a short cord. E/o occipital encephalocele with kyphoscoliotic deformity. NT could not be made out.	Not done

6	21	11-12	Absent calvarium with floating brain parenchyma. Nuchal edema, herniation of heart, liver, bowel loops, and bladder. The eventrated organs are located in the extraembryonic coelomic cavity. Thin amniotic bands and spinal dysraphism were noted (Figure 5).	Not done

7	24	12-13	Increased NT and omphalocele major (herniated liver & urinary bladder) were noted within the coelomic cavity. Short umbilical cord and suspicious unilateral club foot were noted (Figure 6).	Not done

8	22	12-13	Increased NT and thoracoabdominoschisis with eviscerated heart, liver, and urinary bladder noted within the coelomic cavity. Abnormally positioned lower limbs, kyphoscoliotic deformity, and short umbilical cord (Figure 7).	Not done

9	26	11 ± 1	Increased NT and large median ventral wall defect with eventrated organs (heart & liver) which are adherent to placenta cord could not be made out.	Not done

10	24	Fetus A-11-12, fetus B-12-13	Dichorionic diamniotic live twin pregnancy with fetus B: normal. Fetus A shows herniation of heart, liver, and bladder in the coelomic cavity. Abnormally placed lower limbs and kyphoscoliotic deformity. NT was raised (Figure 8).	

**Table 3:** Differential diagnosis of abdominal wall defects in first trimester.

	Herniated viscera	Herniation site	Umbilical cord	Amniotic membrane	Coelomic space	Fetal mobility	Spine	
Exomphalos	Liver and bowel	Base of Umbilical cord	Free floating	Continuous. Fused with Chorion	Obliterated	Normal	Normal/kyphoscoliosis	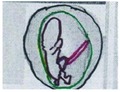

Gastroschisis	Bowel	Amniotic cavity	Free floating	Continuous. Fused with Chorion	Obliterated	Normal	Normal	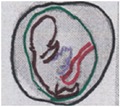

Pentalogy of Cantrell	Heart, liver & bowel	Amniotic cavity	Free floating	Continuous. Fused with Chorion	Obliterated	Normal	Normal	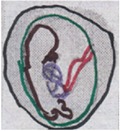

Cloacal exstrophy & OEIS complex	Cloaca	Amniotic cavity	Free floating	Continuous. Fused with Chorion	Obliterated	Normal	Normal/kyphoscoliosis	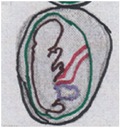

Body stalk anomaly	Liver and bowel	Coelomic cavity	Absent/short cord	Interrupted at the site of herniation	Contain abdominal organ	Stuck through abdominal organ	Kyphoscoliosis	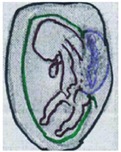

Abdominoschisis with amniotic bands	Liver and bowel	Amniotic cavity	Free floating	Ruptured	Obliterated	Normal	Normal